# Mortality related to drug-resistant organisms in surgical sepsis-3: an 8-year time trend study using sequential organ failure assessment scores

**DOI:** 10.1007/s10096-020-04037-w

**Published:** 2020-09-21

**Authors:** Junichi Yoshida, Tetsuro Tamura, Kazuhiro Otani, Masaaki Inoue, Eiji Miyatake, Toshiyuki Ishimitsu, Chihiro Nakahara, Masao Tanaka

**Affiliations:** Department of Surgery/Chest Surgery, Shimonoseki City Hospital, 1-13-1 Koyo-cho, Shimonoseki, 750-8520 Japan

**Keywords:** Sepsis, Methicillin-resistant *Staphylococcus aureus*, Sequential organ failure assessment score

## Abstract

The difference in sequential organ failure assessment (SOFA) scores from the baseline to sepsis is a known predictor of sepsis-3 outcome, but the prognostic value of drug-resistant organisms for mortality is unexplained. We employed sepsis stewardship and herein report an observational study. Study subjects were patients admitted to the Departments of Surgery/Chest Surgery from 2011 through 2018 with a diagnosis of sepsis and a SOFA score of 2 or more. Our sepsis stewardship methods included antimicrobial and diagnostic stewardship and infection control. We determined the primary endpoint as in-hospital death and the secondary endpoint as the annual trend of the risk-adjusted mortality ratio (RAMR). For mortality, we performed logistic regression analysis based on SOFA score, age, sex, comorbid disease, and the presence of methicillin-resistant *Staphylococcus aureus* (MRSA) and extended-spectrum beta-lactamase inhibitor–producing bacteria. In a total of 457 patients, two factors were significant predictors for fatality, i.e., SOFA score of 9 or more with an odds ratio (OR) 4.921 and 95% confidence interval [95% CI] 1.968–12.302 (*P* = 0.001) and presence of MRSA with an OR 1.83 and 95% CI 1.003–3.338 (*P* = 0.049). RAMR showed a decrease during the study years (*P* < 0.05). Early detection of MRSA may help patients survive surgical sepsis-3. Thus, MRSA-oriented diagnosis may play a role in expediting treatment with anti-MRSA antimicrobials.

## Introduction

Since 2004 in Italy, sepsis stewardship has been defined to integrate the early detection of sepsis and promote education among health service professionals [1]. In 2012, Girardis et al. [2] reviewed six papers on sepsis stewardship programs and stated that early identification and proper management of patients with sepsis were key factors, as did Cavazzuti and others [1]. Thereafter, in 2016, sepsis was newly defined as sepsis-3 using an increase in the sequential organ failure assessment (SOFA) score from the baseline SOFA [3]. This change was based on the predictability of its in-hospital prognosis [4].

To reduce the mortality of patients with sepsis, the guidelines proposed early goal-directed therapy (EGDT), including administration of broad-spectrum antimicrobials in the hour bundles [3]. However, antimicrobial and diagnostic stewardship (AS and DS, respectively) have also been advocated since the turn of the twenty-first century. AS regulates the use of broad-spectrum antimicrobials and DS regulates the correct use of laboratory resources, and the purposes of both are to aid in reducing multidrug-resistant organisms as well as *Clostridioides difficile* infection (CDI) [5]. Thus, AS for the benefit of people in general may defy EGDT for sepsis patients’ survival.

Herein, we attempt to identify drug-resistant organisms and the effect of sepsis stewardship in surgical sepsis-3 using an observational study.

## Methods

Study subjects were patients admitted to the Departments of Surgery/Chest Surgery from 2011 through 2018 with a diagnosis of sepsis and SOFA score of 2 or more. SOFA scores in the absence of patient baseline data were calculated based on the postsepsis status. Likewise, for the computation of respiratory scores, partial arterial pressure of oxygen was extrapolated from percutaneous oxygen saturation using the oxygen dissociation curve [6] because of frequent missing data. Additionally, the fraction of inspiratory oxygen was approximated from the oxygen inhalation flow per minute and the oxygen delivery device [7].

To control drug-resistant organisms, we maintained AS, DS, and infection control. Blood cultures were mandated in response to the prescription of broad-spectrum antimicrobials, which included tazobactam/piperacillin, meropenem, imipenem/cilastatin, cefepime, and panipenem/betamipron. When parenteral vancomycin was prescribed, pharmacists assisted with the dosage plan and therapeutic drug monitoring. From 2011, we used VITEK2 (BioMerieux Japan, Tokyo) for the rapid diagnosis of drug resistance. After 2018, we employed matrix-assisted laser desorption and ionization time-of-flight (MALDI BioTyper; Beckton, Dickinson and Co.), which allowed the rapid identification of species. When *Staphylococcus aureus* was detected from blood specimens before the detection of methicillin-resistant *S. aureus* (MRSA), we advised the attending surgeons to prescribe anti-MRSA antimicrobials. Likewise, when blood culture detected *Escherichia coli* and *Klebsiella pneumoniae* before extended-spectrum beta-lactamase inhibitor–producing organisms (ESBLs) were excluded, we advised the use of cefmetazole or carbapenems. When the organisms were proven to be neither MRSA nor ESBLs, patients underwent de-escalation of antimicrobial therapies.

For the primary endpoint of in-hospital death, we used receiver operating characteristic (ROC) analysis to determine the cutoff points for SOFA and age. The higher values of SOFA and age, sex, common disease, and the presence of MRSA and ESBLs underwent logistic regression analysis for mortality. MRSA and ESBLs were selected because of their prevalence in the country [8]. To see the changes in background factors by year, we used Pearson’s chi-squared test for all the study years to avoid multiple comparisons between an index year and the other 7 years. Statistical significance was determined by two-sided *P* < 0.05.

We determined the secondary endpoints as described hereafter. The first endpoint was the annual trend of mortality of patients with surgical sepsis-3. To adjust mortality for SOFA levels, we chose an international study on the relationship between SOFA and mortality [9], which we defined as the predicted event to calculate the risk-adjusted mortality ratio (RAMR) [10]. For each year, we calculated

$$ \mathrm{RAMR}=\Sigma \left(\left(\mathrm{Observed}\ \mathrm{Event}\right)/n\right)/\Sigma \left(\left(\mathrm{Predicted}\ \mathrm{Event}\right)/n\right) $$

where Observed Event was either 1 for death or 0 for survival and “*n*” was the annual number of patients. Subsequently, a trend graph was generated that depicted observed mortality, RAMR, and SOFA.

As another secondary endpoint, we examined the possible association between organ dysfunction and fatality. SOFA scores of individual organs were denoted as 0 or 1 for values below or above their means, respectively. All of the patients’ SOFA score data underwent logistic regression analysis to identify independent organ failure factors related to the risk of in-hospital death. Then, the same analysis was performed on the data for a subset of patients who underwent surgical operations. To avoid multiple comparisons, *P* values were obtained for multivariate regression analyses alone.

For statistical computation, we used SPSS Statistics V26 (IBM Corporation, Armonk, NY, US).

## Results

Of a total of 457 patients, 109 patients (23.9%) did not survive. Between the survival and in-hospital death groups, the survivors showed a higher rate of gastrointestinal diseases, whereas the nonsurviving patients had an increased rate of chest illness (Table 1). A variety of benign diseases of the gastrointestinal organs, however, were experienced by 153 patients. A total of 165 patients (36.1%) underwent operations, while the other patients were admitted for nonoperative treatments in the Departments of Surgery/Chest Surgery. The annual rate of disseminated intravascular coagulation showed a peak in 2016 (Table 2).

**Table 1:** Background factors of patients analyzed using Pearson’s chi-squared tests (*, two-sided *P* < 0.05). Patients with or without in-hospital deaths. The rate of gastrointestinal diseases was higher in the survival group, whereas that of chest diseases was higher in the mortality group

Death	No	Yes	Total	Chi-squared test
*N*	348	109	457	
Age (range)	75.37 (16–100)	77.94 (39–95)	75.99 (16–100)	0.071
Male rate	0.61	0.61	0.61	1.000
Pneumonia rate	0.22	0.23	0.22	0.791
Chest diseases rate	0.31	0.43	0.34	0.020*
Gastrointestinal diseases rate	0.50	0.38	0.47	0.021*
MRSA^a^ rate	0.12	0.19	0.14	0.054
ESBL^b^ rate	0.07	0.07	0.07	0.832
CDT+^c^ rate	0.03	0.06	0.04	0.156
DIC^d^ rate	0.09	0.15	0.11	0.109

^a^Methicillin-resistant *Staphylococcus aureus*

^b^Extended-spectrum beta-lactamase–producing microbes

^c^*Clostridioides difficile* toxin-positive

^d^Disseminated intravascular coagulation

**Table 2:** Background factors of patients analyzed using Pearson’s chi-squared tests (*, two-sided *P* < 0.05). Annual trends of background factors. The rate of disseminated intravascular coagulation (DIC) peaked in 2016

Year	2011	2012	2013	2014	2015	2016	2017	2018	Total	Chi-squared test
*N*	26	36	75	70	87	66	38	59	457	*P*
Age (range)	78.35 (43–92)	74.47 (20–95)	76.04 (39–98)	78.14 (47–96)	77.41 (38–100)	72.17 (16–93)	77.32 (51–96)	74.56 (19–95)	75.99 (16–100)	0.368
Male rate	0.73	0.61	0.6	0.56	0.62	0.64	0.58	0.58	0.61	0.878
Pneumonia rate	0.23	0.17	0.2	0.23	0.26	0.2	0.13	0.27	0.22	0.700
Chest diseases rate	0.27	0.28	0.35	0.34	0.39	0.32	0.26	0.37	0.34	0.820
Gastrointestinal diseases rate	0.58	0.58	0.43	0.51	0.45	0.48	0.39	0.44	0.47	0.606
MRSA rate	0.23	0.25	0.09	0.17	0.09	0.17	0.05	0.12	0.14	0.092
ESBL rate	0.04	0.08	0.05	0.06	0.03	0.08	0.18	0.08	0.07	0.161
CDT+ rate	0	0.11	0.07	0.06	0.01	0.05	0.03	0	0.04	0.086
DIC rate	0	0	0.12	0.11	0.03	0.17	0.13	0.2	0.11	0.004*

For in-hospital mortality, the ROC analysis revealed a cutoff SOFA score of 9 (area under curve (AUC) 0.641, 95% confidence interval (95% CI) 0.578–0.704), and a cutoff age of 78 (AUC 0.548, 95% CI 0.488–0.607). Values above these cutoff points were observed for 21 (4.6%) and 250 (54.7%) patients, respectively.

For the primary endpoint, multivariate regression analysis showed two significant factors: (1) SOFA higher than 9 with an odds ratio (OR) of 4.921 and 95% CI of 1.968–12.302 (*P* = 0.001) and (2) presence of MRSA with an OR of 1.83 and 95% CI of 1.003–3.338 (*P* = 0.049) (Table 3).

**Table 3:** Univariate and multivariate logistic regression analysis on risk factors for the primary endpoint of in-hospital death

Factors	Univariate analysis		Multivariate analysis		*P*
	OR^a^	95% CI^b^	OR	95% CI	
ESBL^c^	1.069	0.466–2.454	0.916	0.382–2.197	0.844
SOFA^d^	4.66	1.907–11.384	4.921	1.968–12.302	0.001*
Age > = 78	1.438	0.927–2.232	1.477	0.933–2.338	0.096
Male	0.997	0.642–1.548	1.197	0.751–1.906	0.449
MRSA^e^	1.787	1.004–3.181	1.83	1.003–3.338	0.049*
Pneumonia	0.941	0.557–1.591	0.923	0.537–1.589	0.774

^a^Odds ratio

^b^Confidence interval

^c^Extended-spectrum beta-lactamase–producing organism

^d^Score of 9 or more on sequential organ failure assessment

^e^Methicillin-resistant *Staphylococcus aureus*

*P < 0.05 with significance

**Fig. 1:**
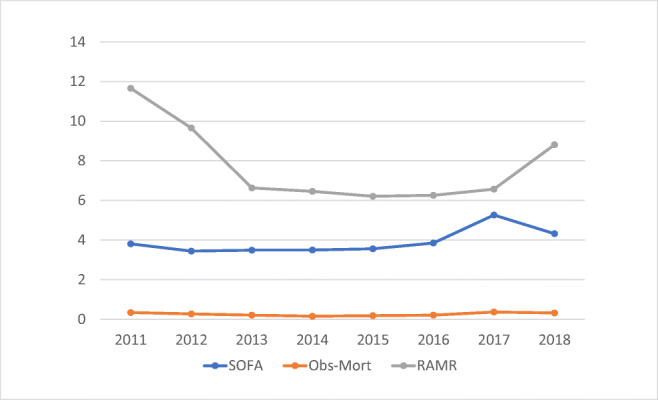
Time trend of mean values of sequential organ failure assessment (SOFA) scores, observed (O_) mortality, and SOFA risk-adjusted mortality ratio (RAMR). All the three variables are dimensionless along the vertical axis. RAMR decreased with statistical significance (two-sided *P* = 0.038, Pearson’s chi-squared test), whereas SOFA (*P* = 0.198) and O_mortality (*P* = 0.092) showed nonsignificant fluctuations

The secondary outcome of the annual trends showed that RAMR decreased by the year 2013 with statistical significance (two-sided *P* = 0.038, Pearson’s chi-squared test), while the observed mortality (*P* = 0.092) and SOFA scores (*P* = 0.198) fluctuated (Fig. 1).

Multivariate regression analysis of the organ components of the SOFA scores for all patients (*N* = 457) revealed that dysfunction of the lungs, blood, heart, and kidney increased risk, while hepatic dysfunction decreased risk for in-hospital mortality (*P* < 0.05) (Table 4). Pulmonary and hematological dysfunction showed high positive odds ratios of 2.553 and 2.376, respectively. Conversely, the probable reason for the decreased risk in patients with liver dysfunction was the effect of infection source control for obstructive cholangitis. For postoperative patients (*N* = 165), multivariate analysis showed that hematologic dysfunction, i.e., thrombocytopenia alone, contributed to mortality (Table 5).

**Table 4:** Univariate and multivariate logistic regression analyses with odds ratios (ORs) for mortality by the components of the sequential organ failure assessment (SOFA) score with an increase of 2 or more. Analysis for all the patients (*N* = 457) in the surgical departments. Patients who had increased values for the components of the lungs, blood, heart, and kidney were at increased risk for mortality, while patients with increased values of the liver component were at reduced risk

Organs	Univariate analysis		Multivariate analysis		*P*
	OR	95% CI^a^	OR	95% CI	
Lungs	2.223	1.416–3.491	2.553	1.533–4.252	< 0.001*
Blood	1.703	1.088–2.665	2.376	1.436–3.930	< 0.001*
Liver	0.617	0.397–0.959	0.6	0.370–0.974	0.039*
Heart	2.423	1.530–3.835	2.001	1.213–3.303	0.007*
Brain	1.456	0.929–2.559	1.333	0.710–2.502	0.371
Kidney	1.846	1.179–2.889	2.098	1.290–3.413	0.007*

^a^Confidence interval

*P < 0.05 with significance

**Table 5:** Univariate and multivariate logistic regression analyses with odds ratios (ORs) for mortality by the components of the sequential organ failure assessment (SOFA) score with an increase of 2 or more. (3-b) Analysis for patients undergoing operations (*N* = 165). Increased values of the blood component increased risk

Organs	Univariate analysis		Multivariate analysis		*P*
	OR	95% CI	OR	95% CI	
Lung	2.487	1.201–5.151	2.961	1.222–7.325	0.076
Blood	3.42	1.608–7.273	5.158	2.162–12.308	< 0.001*
Liver	0.526	0.256–1.078	0.528	0.231–1.206	0.129
Heart	1.456	0,714–2.966	1.157	0.497–2.693	0.735
Brain	1.359	0.541–3.418	1.381	0.469–4.063	0.558
Kidney	1.867	0.914–3.815	2.205	0.995–4.889	0.052

*P < 0.05 with significance

## Discussion

We revealed that increased SOFA scores and the presence of MRSA significantly influenced in-hospital fatality in surgical patients with sepsis-3. The life-saving critical point of SOFA at 9 may serve as an indicator for the triage of patients with sepsis-3 who are at an increased risk of death. Fuchs et al. [11] found that patients in the surgical intensive care unit (ICU) showed a SOFA score of 7 as the critical point of mortality. Vincent and others [9] reported that at a SOFA score of 10, mortality increased by nearly 50% for sepsis patients in general. Thus, patients with SOFA scores of 7 to 10 have poor prognoses.

For immediate assessment of sepsis-3, quick SOFA [3] was defined but was later found to lack accuracy for predicting morbidity in sepsis patients [12, 13]. Falcão et al. [14] stressed that prognostic evaluation in the surgical ICU, including SOFA scores, would help distribute medical resources to the appropriate patients. Herrod and colleagues [15] reported poor predictability of quick SOFA after colonic operations. Thus, SOFA, not quick SOFA, scoring may serve as a practical tool for sepsis stewardship.

Once any patient meets the definition of sepsis-3, the immediate task for clinical care is EGDT. Seymour and others [16] reported that the key element was the early administration of antimicrobials rather than fluid resuscitation. For this purpose, Cavazzuti et al. [1] described sepsis stewardship, which includes the education of health professionals. Despite the fact that early broad-spectrum antimicrobials are indicated [17], their de-escalation is strongly advised [18]. A report by del Pozo [5] described that sepsis stewardship is a cornerstone in preventing CDI as well. Likewise, Garnacho-Montero et al. [19] reported that de-escalation of antimicrobials resulted in a decrease in mortality in surgical sepsis. Sartelli et al. [20] even stated that if surgeons fail to prescribe antibiotics judiciously, they will be deprived of clinical autonomy.

Regarding DS, a position paper from the European Society of Clinical Microbiology and Infectious Diseases in 2018 [21] stated that precision medicine is now needed for the treatment of sepsis. For pathogen-negative sepsis, Lockhart and others [22] reported that more precise use of antimicrobials is indicated. Liesenfeld and others [23] described molecular diagnosis in sepsis, including MALDI spectrometry, serving as a form of precision medicine. In the near future, MALDI spectrometry along with antibiotic susceptibility of patient cultures would provide accurate antimicrobial therapy for sepsis patients [24]. Our study also employed MALDI, but determination of its role awaits future study because of its limited years of use.

Regarding multidrug-resistant organisms, Mangioni and others [25] stressed the importance of triage in sepsis patients. Capsoni et al. [26] described that ESBLs were life-threatening in sepsis patients. Our study disclosed, however, that MRSA was a significant factor in morbidity. Jokinen and others [27], as well, described that MRSA predicted worse outcomes than did methicillin-susceptible *S. aureus*. Ohashi et al. [28] showed, however, that pharmacists’ intervention in MRSA bacteremia improved the survival of sepsis patients. Additionally, Dolin et al. [29] described that broad-spectrum antimicrobials would predispose patients to MRSA infection and thus compromise their prognosis. Thus, MRSA-oriented diagnosis may play a role in expediting the use of anti-MRSA antimicrobials.

For the secondary endpoint of mortality change, we observed a decline in RAMR. Wakeam and others [30] reported that postoperative deep surgical site infection was associated with sepsis. Prescott et al. [31] reported a temporary change in adjusted mortality related to interhospital variability but a persistent decline in sepsis mortality over time. Motzkus and others [32, ] stated that compared with ICU patients admitted from the emergency room, those admitted from the hospital ward had poorer outcomes. Thus, mortality changes and site differences are complex. Other findings of the secondary endpoints that suggest the contribution of pulmonary and hematological organ failure, however, await future validation. The paradoxical finding of hepatic dysfunction being a survival factor, however, indicates the importance of infection source control. On the contrary, Karvellas and others [33] indicated that the delay in biliary decompression and antimicrobials in cholangitis-associated septic shock caused poor outcomes. Thus, biliary drainage and early administration of antimicrobials are pivotal in the management of septic liver dysfunction.

The limitations of our study include the retrospective observation involving historical bias. Specifically, stewardship intervention was a gradual reinforcement and thus did not meet the dichotomous requirement of the logistic regression model. Second, sepsis-related mortality dependent on the SOFA score was adjusted by external resources as reported in an international study, but the validation of the findings is limited [34]. Another consideration may be serial evaluation of the SOFA score, as reported by Ferreira et al. [35]. Third, we used the oxygen dissociation curve for the approximation of oxygen saturation when calculating the respiratory index of SOFA, which should have been obtained from the blood gas analysis. Such analyses, however, had been scarcely documented, whereas oxygen saturation was uniformly available in our patients’ medical records. Furthermore, we considered that such approximation may expedite proper evaluation using SOFA scores better than using quick SOFA [3]. Fourth, the presence of MRSA was not necessarily pathogenic in sepsis patients. Tellor et al. [36], however, described that MRSA colonization as well increased the risk of fatal outcomes in intra-abdominal sepsis.

For statistical analysis of the time trend data, we avoided using multiple comparisons in accordance with a major journal [37], which stresses that *P* values are discouraged to evaluate secondary endpoints. Thus, we refrain from forming definitive conclusions based on the analysis of organ dysfunction components.

We conclude that the presence of MRSA in blood cultures was a significant prognostic factor for mortality in surgical sepsis patients. Thus, MRSA-oriented diagnosis may play a role expediting the use of anti-MRSA antimicrobials.
